# Effects of Content of Soil Rock Fragments on Soil Erodibility in China

**DOI:** 10.3390/ijerph19020648

**Published:** 2022-01-06

**Authors:** Miaomiao Yang, Keli Zhang, Chenlu Huang, Qinke Yang

**Affiliations:** 1 College of Urban and Environmental Sciences, Northwest University, Xi’an 710127, China; mmyang@stumail.nwu.edu.cn; 2 College of Geography, Beijing Normal University, Beijing 100875, China; keli@bnu.edu.cn; 3 College of Tourist (Institute of Human Geography), Xi’an International Studies University, Xi’an 710127, China; nwuhcl@163.com

**Keywords:** soil erosion, soil erodibility, rock fragment, redundancy analysis, elevation

## Abstract

Soil erosion is serious in China—the soil in plateau and mountain areas contain a large of rock fragments, and their content and distribution have an important influence on soil erosion. However, there are still no complete results for calculating soil erodibility factor (K) that have corrected rock fragments in China. In this paper, the data available on rock fragments in the soil profile (RFP); rock fragments on the surface of the soil (RFS); and environmental factors such as elevation, terrain relief, slope, vegetation coverage (characterised by normalised difference vegetation index, NDVI), land use, precipitation, temperature, and soil type were used to explore the effects of content of soil rock fragments on calculating of K in China. The correlation analysis, typical sampling area analysis, and redundancy analysis were applied to analyse the effects of content of soil rock fragments on calculating of K and its relationship with environment factors. The results showed that (1) The rock fragments in the soil profile (RFP) increased K. The rock fragments on the surface (RFS) of the soil reduced K. The effect of both RFP and RFS reduced K. (2) The effect of rock fragments on K was most affected by elevation, followed by terrain relief, NDVI, slope, soil type, temperature, and precipitation, but had little correlation with land use. (3) The result of redundancy analysis showed elevation to be the main predominant factor of the effect of rock fragments on K. This study fully considered the effect of rock fragments on calculating of K and carried out a quantitative analysis of the factors affecting the effect of rock fragments on K, so as to provide necessary scientific basis for estimating K and evaluating soil erosion status in China more accurately.

## 1. Introduction

Soil is a finite natural resource that controls biological, hydrological, erosive, and geochemical cycles [1]. It not only provides survival guarantee for life on earth, but also carries the indispensable material foundation of ecosystems [2]. However, soil erosion has become one of the biggest threats to soil destruction today [3,4]. Soil erosion is one of the world’s environmental problems, and it is also an incentive for many other environmental problems [5]. Due to the influence of China’s special physical geography, social economy, and human factors, land resources are increasingly damaged to meet the needs of human social development, and soil erosion has become more serious [6], making China one of the countries with the most severe soil erosion in the world [7]. According to the bulletin of 2020 Soil and Water Conservation in China, the total area of soil erosion in China is more than 26.9 million km^2^, accounting for 28.14% of the monitored area [8]. Large-scale soil erosion has become an important factor restricting economic development and destroying the health of the ecosystem. Soil erodibility factor (hereinafter referred to as the K), as a quantitative index that characterises the intrinsic properties of soil, is a necessary parameter for soil erosion prediction models [9], and has been widely used in models such as universal soil loss equation (USLE), revised universal soil loss equation (RUSLE), and Chinese soil loss equation (CSLE) [10,11]. Soil erodibility factors are affected not only by soil particle composition, but also by the content of rock fragments (diameter > 2 mm) in the soil [9,10,11,12].

Rock fragments are widely distributed in the soil due to inadequate soil-forming processes [13,14,15,16]. In rocky mountainous areas, there is often a large amount of rock fragments on the surface of the soil (hereinafter referred to as the RFS). For example, in the skeleton soil of Beijing mountainous area, the RFS of the waste grassland is more than 22% [17]. In the karst areas of southwestern China, there are a large number of rocky hills and mountains. The soil in the secondary forest areas of Laoshan Huanglongshan and Qiaoshan Ziwuling in the Loess Plateau contains a lot of rock fragments [18]. Rock fragments cover has been used in agriculture for more than 300 years in arid areas such as Gansu and Ningxia province in China [19]. In addition, due to serious soil erosion, the soil in some areas has serious soil desertification, resulting in a large amount of rock fragments in the soil [20]. The presence of rock fragments changes some physical properties of homogeneous soil, such as the number of macropores, water cross-section, and soil mechanical properties [21]. Furthermore, rock fragment also affects surface runoff and soil erosion by affecting water infiltration [22]. 

Existing studies show that RFS can improve saturated hydraulic conductivity, reduce water evaporation, and increase water infiltration and soil temperature in areas with serious soil erosion [23]. The presence of RFS could protect the soil from raindrop splashes and sealing, reduce surface water flow, reduce runoff, and alleviate soil erosion to a certain extent [24,25,26,27]. Poesen et al. [26] found that an exponential decay function could be used to quantitatively estimate soil erosion reduced by RFS. Meanwhile, RFS can change the biological activity and fertility of soil [28,29], providing a better environmental condition for plant growth [30]. However, the presence of rock fragments in the soil profile (hereinafter referred to as the RFP) reduces saturated hydraulic conductivity and soil permeability rate, leading to a faster response of soil surface runoff, thus increasing surface runoff and sediment yield caused by erosion, and then increasing K [12,31,32]. The distribution characteristics of rock fragments have significant spatial variability, which is significantly affected by slope, elevation, vegetation coverage, climate, and human activities [16,33,34,35,36]. Simanton and Toy [37] noted that hillslope morphology influenced rock fragment cover, finding a logarithmic increase of rock fragment cover with slope angle for hillslopes in semiarid range lands of Arizona, USA. Xia [38] found that rock fragments will be exposed on soil surface because of frequent human activities. Li et al. [33] pointed out that the differences between the spatial patterns of rock fragment and vegetation coverage correlated with land use. The differences of rock fragments distribution characteristics also have important effects on physical and chemical properties of soil and hydrological processes.

China’s complex topographical conditions have caused serious soil erosion, and rock fragments are distributed in a wide area. However, the research on K estimation and evaluation within China did not consider the effect of RFS and RFP on soil erodibility (K) (such as the research of Liang et al. [39] and Teng et al. [40]). Moreover, there are still no complete results for calculating soil erodibility factor (K) that have corrected rock fragments in China. The relationship between the effect of rock fragments on K and influencing factors has not been systematically studied, and the understanding of the effect of rock fragments on K is not comprehensive enough. Research on the effect of rock fragments on K and its influencing factors remains to be carried out in China.

The purpose of this research was to analyze and estimate the effect of content of soil rock fragments on calculating of K in China and to explore the relationship between the effect of rock fragments on K and influencing factors such as elevation, terrain relief, slope, vegetation coverage (NDVI), land use, precipitation, temperature, and soil type, etc., in order to understand the current situation and regular pattern of soil erosion in China more comprehensively and accurately. This research provides a scientific basis for soil erosion prevention and control, soil erosion survey, and application of K. It is also of great significance for wind erosion prediction and hydrological process research.

## 2. Materials and Methods

### 2.1. Data

#### 2.1.1. Data Source

This study was carried out in China. The basic data used in our article include RFS, RFP, and soil type data with a resolution of 250 m downloaded from ISRIC (International Soil Reference and Information Centre) website, elevation and NDVI with a resolution of 250 m, and land use data with a resolution of 30 m downloaded from Resource and Environmental Science and Data Center, as well as 2010–2017 annual average precipitation and temperature site data including meteorological station data of 338 prefecture-level cities downloaded from the National Meteorological Science Data Center (Table 1).

#### 2.1.2. Data Pre-Processing

*Spatial interpolation*: Interpolation is a procedure that depends on the spatial and/or statistical properties of the analysed variable(s) [41]. The inverse distance weighting (IDW) interpolation is the widely used spatial interpolation method, which has the advantages of simple principle and fast calculation, and conforms to the first law of geography. It has been widely used in DEM construction, meteorological analysis, hydrological analysis, and so on [42]. IDW estimates values at un-sampled points by the weighted average of observed data at surrounding points. Thus, this can be defined as a distance reverse function of each point from neighbouring points [43]. That means by using a linear combination of values at a known sampled point, values at un-sampled points can be calculated. IDW relies on the theory that the unknown value of a point is more influenced by closer points than by points further away [42]. 

Regarding the spatial interpolation of meteorological data, Dirks et al. [44] recommended the use of IDW for interpolations for spatially dense networks. In this paper, the annual average total precipitation and annual average temperature were prepared using the IDW method for map generation (Geographic Information System software (GIS) ArcGIS™ 10.5 invented by ESRI (Environmental Systems Research Institute in Redlands, CA, USA) and its extension Geostatistical Analyst) according to the monthly average precipitation and temperature data of 338 cities above the prefecture level in China from 2010 to 2017. Spatial distribution maps of the annual average total precipitation and annual average temperature were constructed according to the coordinates and sample data by using IDW method. 

*Calculation of slope and terrain relief*: Derived from elevation data with a resolution of 250 m in China to obtain terrain relief and slope data with a resolution of 250 m based on the ArcGIS 10.5.

### 2.2. Methods

#### 2.2.1. Calculation of the Effect of Rock Fragments on K

*The effect of RFS on K*: Poesen [26] proposed an algorithm for calculating attenuation coefficients of rock fragment. This method was used to analysis the effect of RFS on K in this study, through calculating the rock fragment reduction coefficient St, which represents the influence coefficient of RFS (Equation (1)).

(1) $$\mathrm{St}=1-e^{-0.04\left(R_{c}-10\right)}$$

where R_c_ is the rock fragments on the surface of the soil (%), and the larger St is, the more obvious the weakening effect of RFS on K.

*The effect of RFP on K*: The USLE algorithm, proposed by Wischmeier [11,45], is a commonly used algorithm to calculate the K [12,46]. This study used the USLE-K algorithm as an example to discuss the effect of RFP on K. The equation for the USLE-K is

(2) $$K_{\mathrm{USLE}}=\frac{{2.1\times 10}^{-4}M^{1.14}\left(12-\mathrm{OM}\right)+3.25\left(s-2\right)+2.5\left(p-3\right)}{100}\times 0.1317$$

(3) $$M=(M_{\mathrm{silt}}+M_{\mathrm{vfs}})\times (100-M_{c})$$

where M_c_ is the clay content (%), M_silt_ is the silt content (%), M_vfs_ is the very fine sand content (%), OM is the soil organic carbon content (%), s is the soil structure class, and p is the soil profile permeability class.

The K affected by RFP (Kc) and K without considering the rock fragments (Kf) were calculated by Equations (4) and (5). Calculate the difference between the Kc and Kf to obtain the influence coefficient of RFP (K_cf_). The larger the K_cf_, the more obvious the enhancement effect of the RFP on K.

(4) $$\mathrm{Kf}=\frac{2.1\times 10^{-4}M^{1.14}\;\left(12-\mathrm{OM}\right)+3.25\;(s-2)+K_{\mathrm{pf}}}{100}\times 0.1317$$

(5) $$\mathrm{Kc}=\frac{2.1\times 10^{-4}M^{1.14}\;\left(12-\mathrm{OM}\right)+3.25\;(s-2)+K_{\mathrm{pc}}}{100}\times 0.1317$$

(6) $$K_{pf}=2.5(P_{f}-3)$$

(7) $$K_{pc}=2.5(P_{c}-3)$$

(8) $$K_{\mathrm{cf}}=\mathrm{Kc}-\mathrm{Kf}$$

K_pf_ is the permeability coefficient that does not consider the effect of RFP, and K_pc_ is the permeability coefficient that considers the effect of RFP. P_f_ is the permeability class without considering the RFP; P_c_ is the permeability class considering the RFP.

*The effect of both RFP and RFS on K*: Multiplying St and Kc to calculate the K considering the effect of both RFP and RFS (K_cs_).

(9) $$K_{\mathrm{cs}}=\left(1-\mathrm{St}\right)\times \mathrm{Kc}$$

Calculate the difference between Kf and K_cs_ to obtain the comprehensive influence coefficient of both RFP and RFS (K_f–cs_).

(10) $$K_{f–\mathrm{cs}}=\mathrm{Kf}-K_{\mathrm{cs}}$$

The effect of rock fragments on K (hereinafter referred to as the E_R_K_) is expressed by the influence coefficient of RFS (St), the influence coefficient of RFP (K_cf_), and the comprehensive influence coefficient of both RFP and RFS (K_f–cs_).

#### 2.2.2. Sampling Statistics and Typical Sampling Area Analysis

E_R_K_ (St, K_cf_, K_f–cs_) and terrain relief, slope, elevation, NDVI, precipitation, and temperature were sampled at the central point of 1:50,000 standard map (latitude difference is 10′, longitude difference is 15′). A total of 638 samples were collected in China, which were evenly distributed and had certain representativeness. The relationship between the E_R_K_ and terrain relief, slope, elevation, NDVI, precipitation, and temperature was obtained by fitting analysis of the values of sampling points. The correlation between influencing factors and E_R_K_ was compared, and their significance was tested. When analysing the effect of land use and soil type, the mean values of E_R_K_ of different land use and soil type were counted, and the reasons for the high and low values were analysed. For the selection of typical sampling areas, from the low value of influencing factors to the high value, we selected five small sampling areas with different gradients (very low, low, medium, high, and very high) of terrain relief, slope, elevation, NDVI and land use, and their regular patterns and correlations were analysed to discuss the influence of different factors on E_R_K_.

#### 2.2.3. Redundancy Analysis

Redundancy analysis (RDA) is a well-known multivariate method that models the information flow between two datasets by maximising the redundancy index between explanatory and response variables; thus, RDA measures the effect of the explanatory dataset on the response dataset [47]. RDA describes the relationships between datasets by finding a linear combination of the explanatory variables that explain the most variance of the response variables. RDA is a kind of constrained sequencing, similar to multiple regression analysis, which is a direct gradient analysis method. It neither combines several variables into a virtual complex variable nor simply analyses groups of variables. Its biggest advantage is that it can independently maintain the contribution rate of each explanatory variable. The statistical characteristics of a single explanatory variable can be described under different combinations of explanatory variables, and then the choice of explanatory variables can be decided. 

RDA’s conceptual model was already described by Wollenberg [48] and has been widely used in fields such as geology [49,50], ecology [51], and psychology [52]. RDA was used to evaluate the effect of influencing factors on spatial differentiation of E_R_K_, and to identify the main predominant factor affecting spatial variation of E_R_K_ in this study. Influencing factors were selected as indicators to measure the effect of rock fragments, and St, K_cf_, and K_f–cs_ values were selected as response variables in RDA analysis. Eight influencing factors, namely, terrain relief, elevation, slope, NDVI, land use, precipitation, temperature, and soil type, were selected as explanatory variables. The RDA was analysed using CANOCO 5.0 software. 

## 3. Results

### 3.1. The Effect of Rock Fragments on K (E_R_K_)

**Figure 1:**
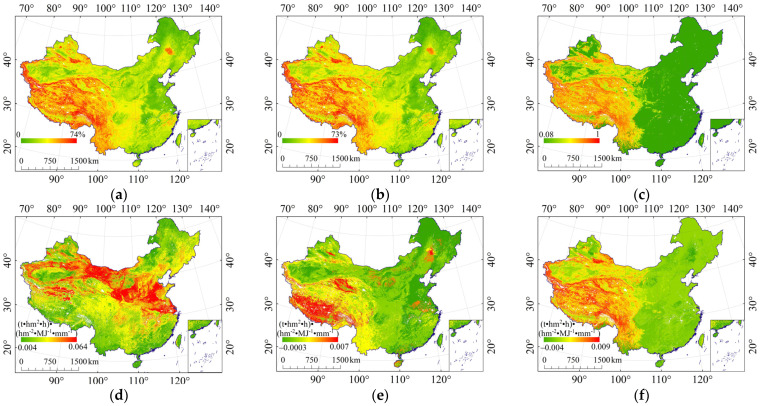
Content of rock fragments and its effects. (**a**) RFP; (**b**) RFS; (**c**) St; (**d**) Kf; (**e**) K_cf_; (**f**) K_f__–__cs_.

The average value of the influence coefficient of RFS (St) was 0.292 (Figure 1c), which has a significant weakening effect on K, reducing K by 0.0094 (t·hm^2^·h)·(hm^−2^·MJ^−1^·mm^−1^). The reason is that RFS slows down raindrop splashes and reduces surface water flow, reducing runoff, thus alleviating soil erosion [9,34,36]. RFP increases K by 0.0011 (t·hm^2^·h)·(hm^−2^·MJ^−1^·mm^−1^) on average in China, because the increase of RFP reduces soil porosity, thus reducing infiltration and increasing surface runoff, soil erosion, and sediment yield [20,21,26,31]. The effect of both RFP and RFS reduced K by an average of 0.0013 (t·hm^2^·h)·(hm^−2^·MJ^−1^·mm^−1^), which occurs in Yunnan–Guizhou Plateau, Qinghai–Tibet Plateau, and Tianshan Mountains with high rock fragment coverage. The protective effect of RFS is greater than RFP in those areas. However, in the eastern multi-plain area, the effect of both RFS and RFP is weak. In general, the effect of RFS on the whole is greater.

### 3.2. Relationship between the Effect of Rock Fragments on K (E_R_K_) and Topography

#### 3.2.1. Relationship between the Effect of Rock Fragments on K (E_R_K_) and Terrain Relief

**Figure 2:**
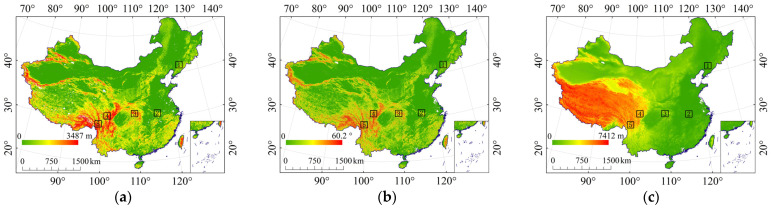
Topographic data and typical sampling areas. (**a**) Terrain relief and its sampling areas; (**b**) slope and its sampling areas; (**c**) elevation and its sampling areas.

**Figure 3:**
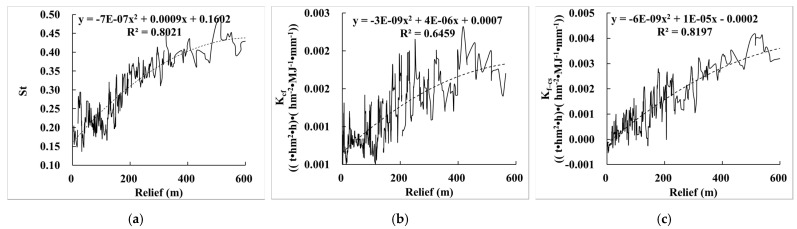
Relationship between E_R_K_ and terrain relief. (**a**) The relationship between St and terrain relief; (**b**) the relationship between K_cf_ and terrain relief; (**c**) the relationship between K_f–cs_ and terrain relief.

By comparing the relationship between E_R_K_ and terrain relief (Figure 2a), we found that St, K_cf_, and K_f–cs_ were significantly correlated with terrain relief, with correlation coefficients of 0.802, 0.646, and 0.820, respectively (Figure 3), indicating a high correlation between E_R_K_ and terrain relief. St, K_cf_, and K_f–cs_ increase with the increase of terrain relief, indicating that rock fragment has greater influence on soil erosion with the increase of terrain relief. The reason is that areas with large terrain relief (Figure 2a) have higher terrain (Figure 2c) mainly distributed in mountains and some hilly areas. The soil parent material in these areas is mostly bedrock, and the degree of soil development is low, which makes the RFS and RFP are large (Figure 1a,b), and therefore it has a greater impact on soil erosion (Figure 1).

#### 3.2.2. Relationship between the Effect of Rock Fragments on K (E_R_K_) and Slope

**Figure 4:**
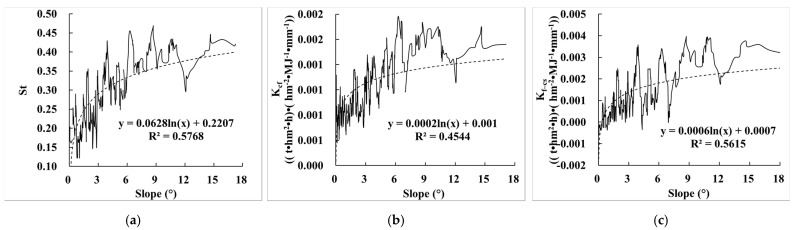
Relationship between E_R_K_ and slope. (**a**) The relationship between St and slope; (**b**) the relationship between K_cf_ and slope; (**c**) the relationship between K_f–cs_ and slope.

Comparing the relationship between E_R_K_ and slope, we found that St, K_cf_, and K_f–cs_ were all significantly correlated with slope, and the correlation coefficients were 0.577, 0.454, and 0.562, respectively (Figure 4). When the slope increased, St, K_cf_, and K_f–cs_ also showed an upward trend. The reason was that the greater the slope, the more mountains and grassland were distributed, and they were more affected by rock fragments. However, when the slope was greater than 6°, the upward trend of St, K_cf_, and K_f–cs_ slowed down significantly. The reason was that areas with a slope greater than 6° are mainly distributed on the border of the Qinghai–Tibet Plateau, as shown in the red area in Figure 2b. These places have better hydrothermal conditions, higher vegetation coverage, and well-developed soil. Moreover, the content of rock fragments in these places is also within a certain threshold range, and therefore the trend of E_R_K_ will tend to be gentle and will not increase indefinitely only because of the increase in slope.

#### 3.2.3. Relationship between the Effect of Rock Fragments on K (E_R_K_) and Elevation

**Figure 5:**
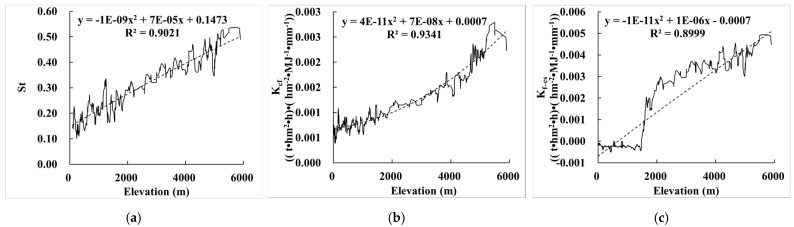
Relationship between E_R_K_ and elevation. (**a**) The relationship between St and elevation; (**b**) the relationship between K_cf_ and elevation; (**c**) the relationship between K_f–cs_ and elevation.

Comparing the relationship between E_R_K_ and elevation, we found that St, K_cf_, and K_f–cs_ were all significantly correlated with elevation, and the correlation coefficients were 0.902, 0.934, and 0.900, respectively (Figure 5). When the elevation increased, St, K_cf_, and K_f–cs_ also showed an upward trend, and the correlation was extremely high. The reason was that in mountainous areas with large terrain relief and slopes, such as the Qinghai–Tibet Plateau, Tianshan Mountains, and Himalayas, there are mainly distributed relatively infertile soils such as Leptosols and Cryosols, with large content of rock fragments. Moreover, the hydrothermal conditions at different elevations also have an indirect impact on the physical and chemical properties of the soil, and thus the higher the elevation, the greater the effect on soil erosion. 

#### 3.2.4. Typical Sampling Areas of Topography

**Figure 6:**
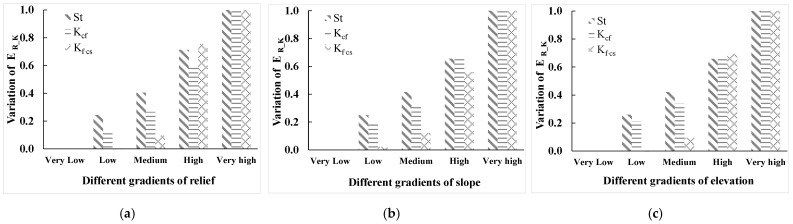
Mean values of E_R_K_ in different typical sampling areas. (**a**) Mean values of E_R_K_ in different terrain relief regions; (**b**) mean values of E_R_K_ in different slope regions; (**c**) mean values of E_R_K_ in different elevation regions.

Five typical sampling areas with different gradients (very low, low, medium, high, and very high) of terrain relief, slope, and elevation were selected, as shown in Figure 2. By calculating the mean values of St, K_cf_, and K_f–cs_ in different regions (Figure 6), we found that the mean values of St, K_cf_, and K_f–cs_ gradually increased with the increase of terrain relief, slope, and elevation, which is consistent with the regular pattern mentioned above. This means that when calculating the effect of rock fragment on K, terrain relief, slope, and elevation factors need to be considered.

### 3.3. Relationship between the Effect of Rock Fragments on K (E_R_K_) and Land Cover

#### 3.3.1. Relationship between the Effect of Rock Fragments on K (E_R_K_) and Land Use

**Figure 7:**
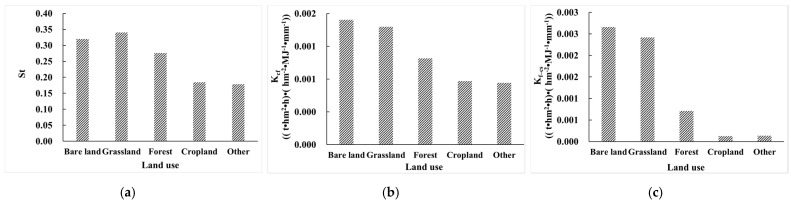
Mean values of E_R_K_ in different land use regions. (**a**) Mean values of St in different land use regions; (**b**) mean values of K_cf_ in different land use regions; (**c**) mean values of K_f–cs_ in different land use regions.

Analysing the average values of St, K_cf_, and K_f–cs_ of bare land, grassland, forest, cropland, and other land, we found that rock fragment had the greatest effect on bare land, followed by grassland and forest, and had the least effect on cropland (Figure 7). The reason is that cropland is usually distributed in relatively flat terrain. The slope and terrain relief of cropland are small—the mean values were 2.534° and 115.965 m, respectively (Table 2). Bare land areas are usually arid, with weak biological activities, and strong winds lead to more content of rock fragments in the soil. The slope and terrain relief of forest and grassland are relatively large. In general, the effect of rock fragments on bare land, grassland, and forest is greater, and the effect of rock fragments on cropland is relatively small (Table 2).

#### 3.3.2. Relationship between the Effect of Rock Fragments on K (E_R_K_) and NDVI

**Figure 8:**
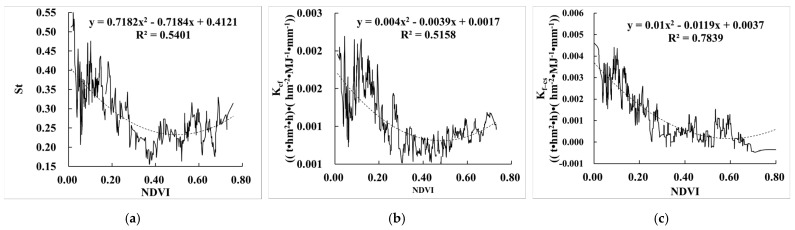
Relationship between E_R_K_ and NDVI. (**a**) The relationship between St and NDVI; (**b**) the relationship between K_cf_ and NDVI; (**c**) the relationship between K_f–cs_ and NDVI.

**Figure 9:**
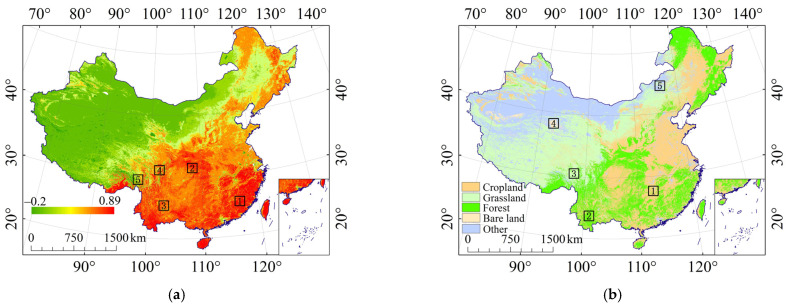
The land cover data and typical sampling areas. (**a**) The NDVI and its sampling areas; (**b**) the land use and its sampling areas.

Figure 8 shows that the relationship between NDVI and E_R_K_ generally presented a concave quadratic curve. The minimum value was about 0.4. When the NDVI was less than 0.4, the larger the NDVI, the smaller the St, K_cf_, and K_f–cs_, indicating the smaller impact of NDVI on rock fragments. The reason is that the areas with NDVI less than 0.4 were mainly distributed in the western regions with lower coverage grassland (Figure 9a). The NDVI increased from northwest to southeast, but the content of rock fragments showed the opposite trend, and therefore St, K_cf_, and K_f–cs_ all showed a downward trend. When the NDVI was greater than 0.4, the three figures (Figure 8a–c) all had inflection points, and St, K_cf_, and K_f–cs_ all showed an upward trend. The possible reason was that the areas with small NDVI were mainly cropland, and the content of rock fragments were both small and the effect was small. However, the areas with larger NDVI were mainly distributed in the steep low hills with larger content of rock fragments, and thus the effect was greater.

#### 3.3.3. Typical Sampling Areas of Land Cover

**Figure 10:**
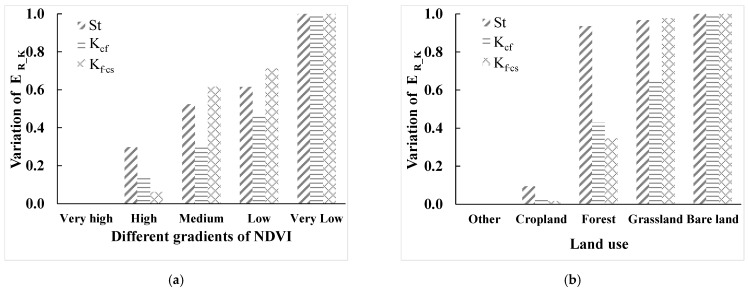
Mean values of E_R_K_ in different typical plots. (**a**) Mean values of E_R_K_ in different NDVI regions; (**b**) mean values of E_R_K_ in different land use regions.

Five typical sampling areas with different gradients (very low, low, medium, high, and very high) of NDVI (Figure 9a) were selected to calculate the mean values of St, K_cf_, K_f–cs_ and NDVI. It was found that the higher the mean value of NDVI in the selected sampling area, the smaller the mean variation of St, K_cf_, and K_f–cs_ (Figure 10a). It shows that the higher the vegetation coverage, the more protective effect on the soil, and the effect of rock fragments on K will be reduced. In this research, a typical sampling area of bare land, cropland, forest, grassland, and other land was selected (Figure 9b), and the mean values of St, K_cf_, and K_f–cs_ were calculated. It was found that when the selected typical sampling area was cropland, the mean variation of St, K_cf_, and K_f–cs_ were small; when the selected typical sampling area was grassland, the mean values of St, K_cf_, and K_f–cs_ varied greatly (Figure 10b), which is consistent with the regular pattern mentioned above. Therefore, the effect of NDVI and different land use should be considered when considering the effect of rock fragments on K.

### 3.4. Relationship between the Effect of Rock Fragments on K (E_R_K_) and Soil Type

This study calculated the average values of St, K_cf_, and K_f–cs_ under different soil types, finding that when the soil types were Leptosols, Cryosols, Gypsisols, Solonchaks, Gypsisols, Luvisols and Calcisols (Table 3), the average values of St, K_cf_, and K_f–cs_ were higher, indicating that the rock fragment has a greater effect on K in this condition. The reason is that this kind of soil is mainly distributed in the west of China. In arid or semi-arid environment, it is mostly shallow soil, which has higher soluble salt content and secondary lime accumulation. Shrubs, grasslands, and grazing are mostly located in these places, and the content of rock fragments are higher, thus the greater the effect on E_R_K_. When the soil types were Fluvisols, Phaeozems, Chernozems, Kastanozems, Cambisols, Cambisols and Alisols (Table 4), the values were lower, indicating that the rock fragment had less effect on K in this condition. The reason was that this kind of soil is mainly distributed in low-lying and humid places, with higher NDVI, richer nutrition, deeper soil, and less content of rock fragments, and therefore it has less effect on E_R_K_.

### 3.5. Relationship between the Effect of Rock Fragments on K (E_R_K_) and Climate

#### 3.5.1. Relationship between the Effect of Rock Fragments on K (E_R_K_) and Temperature

**Figure 11:**
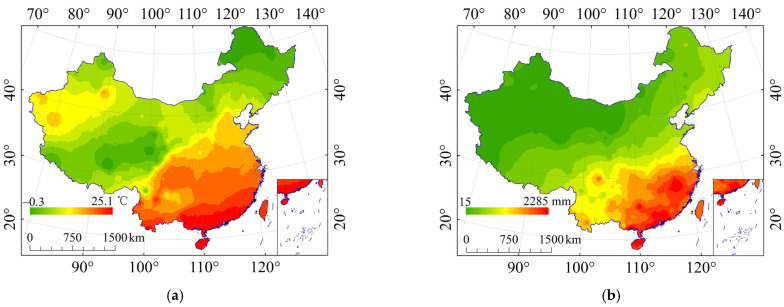
Temperature and precipitation interpolated by IDW method. (**a**) The annual average temperature; (**b**) the annual average total precipitation.

**Figure 12:**
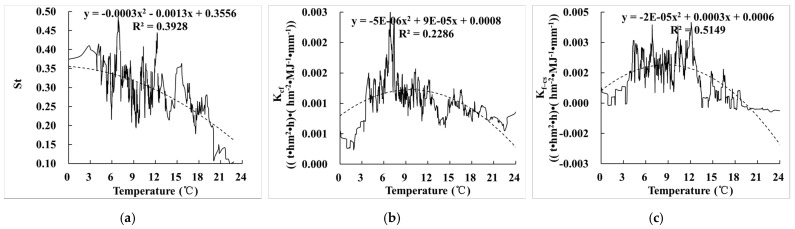
Relationship between E_R_K_ and temperature. (**a**) The relationship between St and temperature; (**b**) the relationship between K_cf_ and temperature; (**c**) the relationship between K_f–cs_ and temperature.

Compared with the relationship between E_R_K_ and temperature (Figure 11a), St, K_cf_, and K_f–cs_ were significantly correlated with temperature, with correlation coefficients of 0.393, 0.229, and 0.515, respectively; the overall trend of St, K_cf_, and K_f–cs_ decreased with the increase of temperature (Figure 12). The reason was that temperature changes soil structure mainly through affecting microbial community, which has great influence on soil spatial distribution and soil physical and chemical properties, thus affecting soil erodibility. The temperature gradually decreases from northwest to southeast, which is opposite to the content of rock fragments and slope distribution. The higher the temperature, the better the hydrothermal conditions, the gentler the terrain, and the better the soil conditions; therefore, the effect of rock fragments will be relatively small. 

#### 3.5.2. Relationship between the Effect of Rock Fragments on K (E_R_K_) and Precipitation

**Figure 13:**
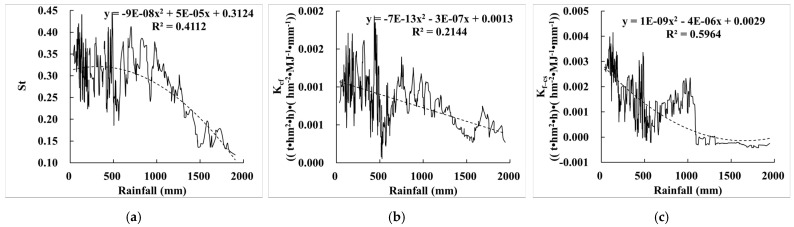
Relationship between E_R_K_ and precipitation. (**a**) The relationship between St and precipitation; (**b**) the relationship between K_cf_ and precipitation; (**c**) the relationship between K_f–cs_ and precipitation.

Compared with the relationship between E_R_K_ and precipitation (Figure 11b), St, K_cf_, and K_f–cs_ were significantly correlated with precipitation, with correlation coefficients of 0.411, 0.214, and 0.596, respectively; the overall trend of St, K_cf_, and K_f–cs_ decreased with the increase of precipitation (Figure 13). The reason is was the areas with high precipitation are mainly distributed in the southeast of China, where the terrain is low and NDVI is high. The soil types are mainly Fluvisols, Phaeozems, and Chernozems, etc. This kind of soil is deep and nutritive, and has less content of rock fragments, and therefore it has less influence on E_R_K__._

### 3.6. Impact of Influencing Factors on the Effect of Rock Fragments on K (E_R_K_)

In order to further clarify the quantitative impact of influencing factors on E_R_K_, this research selected St, K_cf_, and K_f–cs_ values as the response variables in the RDA analysis; eight environmental factors, namely, soil type, elevation, slope, terrain relief, land use, NDVI, precipitation, and temperature were used as explanatory variables. The RDA analysis results showed that eight influencing factors can explain 75.98% of the total information about the spatial variation of E_R_K_; the first axis explained the variation information by 75.98%, and the second axis had no explanation for the variation information. On the axis, the correlation between the response variable and the explanatory variable was 87.17%, and the correlation on the second axis was 49.25%.

**Figure 14:**
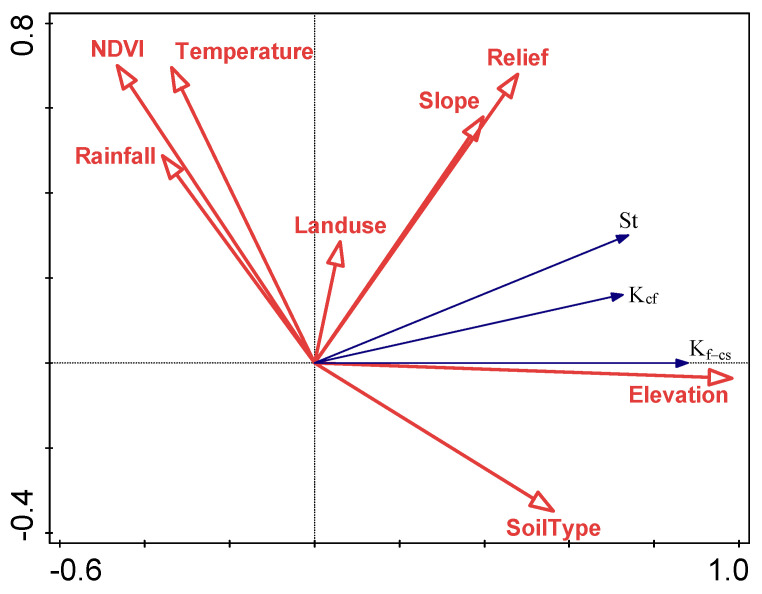
Redundancy analysis (RDA).

In the sequence diagram of RDA (Figure 14), the length of each influencing factor arrow indicates the relative magnitude of the influencing factor’s explanation of E_R_K_. The angle between the two arrows indicates the degree of correlation between the two. When the angle is less than 90°, it indicates that the relationship between the two is positively correlated; when the angle is between 90° and 180°, it indicates that the two are negatively correlated. When it is 90°, it indicates that there is no correlation between the two. Therefore, according to the result of RDA, the values of E_R_K_ were negatively correlated with temperature, precipitation, and NDVI. The values of E_R_K_ were positively correlated with elevation, slope, terrain relief, land use, and soil type. This indicates that there is a correlation between the selected eight influencing factors and E_R_K_. It can be seen from the length of each factor arrow that elevation has the largest amount of explanation, followed by terrain relief, NDVI, slope, soil type, temperature, and precipitation, and land use had a smaller amount of explanation. 

On the basis of the above correlation analysis results, we found that all influencing factors were correlated with the values of St, K_cf_, and K_f–cs_, but the highest correlation was elevation, which was able to explain 74.7% of the values of St, K_cf_, and K_f–cs_, indicating that the elevation is the main predominant factor affecting the spatial variation of E_R_K_, followed by terrain relief, NDVI, slope, soil type, temperature, and precipitation. Therefore, clarifying the spatial distribution of various influencing factors and discussing the variation of the values of St, K_cf_, and K_f–cs_ are of great significance for estimating the soil erodibility factor (K).

## 4. Discussion

### 4.1. The Source and Classes of Rock Fragments

The rock fragment in the soil is a product of weathering and disintegration of rocks; rock fragments may be produced in chemical weathering and root penetration processes that transform rocks into soil [53]; tectonic movement [54]; physical weathering, landslides, and debris flows [55]; and water selective erosion of soil fine particles [37].

Particle sizes of rock fragments are divided into seven classes in the USDA (United States Department of Agriculture) [56]. For simulated rainfall experiments [57] and typical studies of larger particle sizes in small areas [58], the particle size can be distinguished into varied classes. However, for regional calculations and analysis, there is no available data yet. Thus, this study only considers the rock fragments 2 mm in diameter or larger [56].

Due to the lack of a complete set of the coverage of rock fragment data except that rock fragment in special areas or under specific scenarios was reported in some special studies (e.g., Qian et al. [59]; Cao et al. [60]), the data of RFS and RFP used in this paper are SoilGrid dataset from ISRIC (International Soil Reference and Information Centre) data. This dataset provides global predictions for coarse fragments at depths of 0 and 15 cm. Predictions were based on around 150,000 soil profiles used for training and a stack of 158 remote sensing-based soil covariates, which were used to fit an ensemble of machine learning methods [61]. These data have high accuracy and availability. It is expected that in the future, on the basis of high-resolution imaging and UAV (unmanned aerial vehicle) technology, it is possible to distinguish particle sizes in typical areas and analyse the effects of different particle sizes on the erodibility (K factor) or the cover and management factor.

### 4.2. The Influencing Factors of the Effect of Rock Fragment on K

In China, elevation has the greatest influence on St, K_cf_, and K_f–cs._ The reason is that the terrain in China varies greatly, and the elevation is distributed in a three-step pattern [62]. The first step is the Qinghai–Tibet Plateau and the Qaidam Basin, with an average altitude of more than 4000 m. The second step is distributed with large basins and plateaus, with an average elevation between 1000 and 2000 m. The third step covers a vast plain with hills and low mountains in between, including the northeast plain; the North China plain; the plains of the middle and lower Yangtze River; and the hills of Liaodong, Shandong, and Southeast, with elevations below 500 m. Areas with higher elevation are more arid and colder, dominated by physical weathering, and the soil-forming process is weak. Therefore, the soil surface has large content of rock fragments, and the impact of rock fragment is also greater. 

Studies have shown that differences in the size, shape, void type, and particle size of rock fragment have important effects on soil physical and chemical properties, hydrological processes, etc. [26,63,64], thereby affecting soil erosion. The impact of rock fragment is related to tillage measure, soil depth, topographic curvature, physical and chemical properties of parent materials, etc. [53,65,66,67]. However, it is difficult to obtain and apply these data in the whole country. In the future, the environmental factor data and the content of rock fragments can be measured for further discussion when analysing in small watershed, so as to obtain more accurate results of soil erosion evaluation.

### 4.3. The Effect of Rock Fragments on Regional Soil Erosion Evaluation

**Figure 15:**
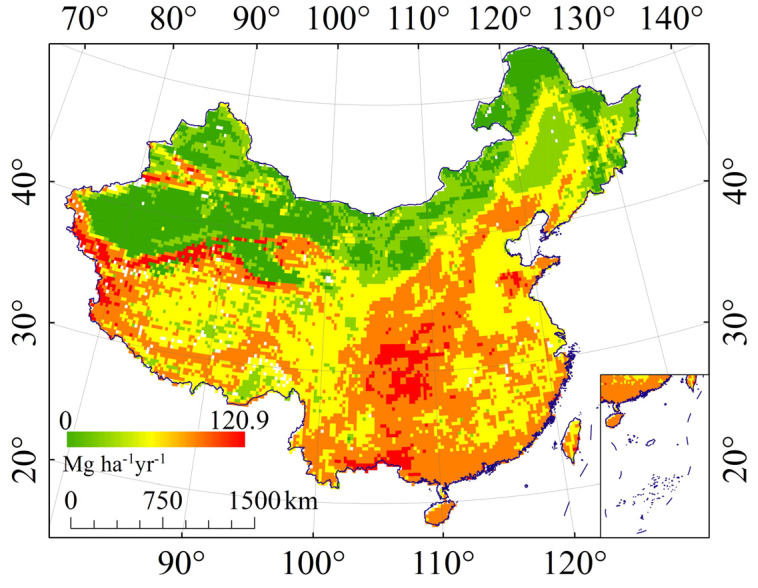
Soil erosion map (Borrelli et al. [46]).

The effect of rock fragments should be fully considered in the calculation of K. If the effect of RFS is not taken into account, the rate of soil erosion is overestimated. If the effect of RFP is not taken into account, the rate of soil erosion is underestimated. Thus, if the effect of rock fragments not considered, the analysis and evaluation of soil erosion obtained will not be accurate. Take the Yunnan–Guizhou Plateau, Qinghai–Tibet Plateau, the Himalayas, and Karakoram Mountains as an example, these areas have high elevation and high terrain relief. The surface is dominated by physical weathering, with large content of rock fragments (Figure 1), and soil erosion is relatively weak. However, the soil erosion evaluation results of Borrelli et al. [46] (Figure 15) show that the soil erosion rate in Himalayas and Karakoram Mountains is significantly higher than that of neighbouring areas. Wang et al. [68] found that the Yunnan–Guizhou Plateau is a hotspot of erosion in southwestern China. In the study of Teng et al. [69], the soil erodibility factor in the eastern part of the Qinghai–Tibet Plateau was overestimated. We suspect that the possible reason was that the effect of rock fragments is not fully considered in their research, and the analysis and evaluation of soil erosion were not complete and accurate. This study fully considered the impact of rock fragments in the calculation of soil erodibility factors, carrying out a quantitative analysis of the influencing factors of the effect of rock fragments on K. Therefore, in the systematic soil erosion evaluation in China, especially in areas with large slopes and insufficient soil water and heat conditions, as well as deserts with low rainfall, the impact of rock fragments should be fully considered in order to have a more comprehensive and accurate understanding of the intensity and spatial differentiation of water erosion in China. 

As for the influencing factors of the effect of rock fragments on K, Zhu and Shao [36] showed that rock fragments were mainly distributed in areas with large slope. Marshall et al. [13] and Nyssen et al. [35] found that rock fragments were more distributed in areas with high altitude and relatively cold and arid mountains and deserts. Although the conclusion of this study was consistent with their understanding, they ignored the influence of land use, soil type, and climate. However, the difference between spatial pattern of rock fragment and vegetation cover is correlated with land use factors [33]. The distribution characteristics of rock fragment are also affected by physical and chemical properties, temperature, and rainfall of parent material [53]. Thus, this study quantified and analysed a variety of influencing factors and can help us understand the effect of rock fragment on K more comprehensively.

### 4.4. Implications for Production Practice

Gravel and sand mulched field (hereinafter referred to as the GSMF) is a kind of farmland covered with a layer of rock fragments on the soil surface. It is a unique drought-resistant farming form in the arid and semi-arid areas of northwest China with annual precipitation of 200–300 mm, and it has a history of more than 300 years [19]. GSMF are mainly distributed in the central part of Gansu Province, as well as parts of Qinghai, Xinjiang, and Ningxia provinces [70]. Rock fragment covering technology has been widely used in the field of agricultural production because of its significant effects of water storage, temperature, and yield increase. Rock fragments on the surface of farmland are beneficial in improving hydrothermal condition and yield. They allow water to infiltrate and store in the soil, effectively maintaining soil moisture and temperature, and can significantly increase crop yields [71,72,73]. Farmers are generally reluctant to remove smaller rock fragments from their land [35] because they believe that they have a positive effect on soil moisture retention and protection of topsoil from erosion. The research of Li et al. [28] showed that rock fragment provides a better growth environment for plant growth in the Loess Plateau. The experiment of Pang Lei et al. [19] showed that soil moisture and the soil microbial quantity all increase when mulched by rock fragment. It is especially beneficial to actinomycete when mulched by bigger diameter gravels. It is optimum for microbial developing when the thickness of gravel-sand is in the range of 7–9 cm. However, these understandings have not been fully considered in soil erosion survey and research in China.

## 5. Conclusions

On the basis of the data of rock fragments and eight environmental factors in China, we used correlation analysis, typical sampling area analysis, and redundancy analysis to explore the effect of content of soil rock fragments on calculating of soil erodibility (K) and its influencing factors. The major conclusions are as follows:

(1) The rock fragments in the soil profile (RFP) increased soil erodibility (K) by 0.0011 (t·hm^2^·h)·(hm^−2^·MJ^−1^·mm^−1^), the rock fragments on the surface (RFS) of the soil reduced soil erodibility (K) by 0.0094 (t·hm^2^·h)·(hm^−2^·MJ^−1^·mm^−1^), and the effect of both RFP and RFS reduced soil erodibility (K) by 0.0013 (t·hm^2^·h)·(hm^−2^·MJ^−1^·mm^−1^).
(2) This effect of rock fragments had the highest correlation with elevation, followed by terrain relief, vegetation coverage (NDVI), slope, soil type, temperature, and precipitation. The higher the elevation, terrain relief, and slope, the greater the effect of rock fragments on K, and the smaller the precipitation, temperature, and vegetation coverage (NDVI), the less the effect of rock fragments on K. When the soil types were Leptosols, Cryosols, Gypsisols, Solonchaks, Gypsisols, Luvisols, and Calcisols, the influence was greater. The effect of rock fragments on K (E_R_K_) had little correlation with land use.
(3) The results of RDA analysis showed that the elevation was the main predominant factor affecting E_R_K_, and the elevation had the greatest influence on St, K_cf_, and K_f–cs._ The reason is that the topography of China varies greatly, with high elevation mountains and plateaus being widely distributed. These mountainous and plateau areas are usually cold and dry, and the soil erosion is mainly wind erosion. Due to the weak soil-forming process, the content of rock fragments is large, and the effect of rock fragments is also great.

In the systematic evaluation of soil erosion in China, the effect of rock fragments should be fully considered, and the effect of rock fragments should be paid more attention to the higher elevation areas such as Qinghai–Tibet Plateau, Yunnan–Guizhou Plateau, and Tianshan Mountains, so as to have a more comprehensive and accurate understanding of the current situation and regular pattern of soil erosion in China.

## Figures and Tables

**Table 1:** Datasets used in this study.

Data	Source	Resolution
RFS ^1^	Download from ISRIC ^4^	250 m
RFP ^2^		
Soil type ^3^		
NDVI	Resource and Environmental Science and Data Center ^5^	250 m
Land use		30 m
Elevation		250 m
Terrain relief	Derived from elevation	250 m
Slope		
Annual average precipitation from 2010 to 2017	Download from National Meteorological Science Data Center ^6^	Meteorological station data of 338 prefecture-level cities
Annual average precipitation from 2010 to 2017		

Note: ^1^ RFS—rock fragments on the surface of the soil; ^2^ RFP—rock fragments in the soil profile; ^3^ soil type—refer to the world reference base for soil resources based on food and Agriculture Organisation of the United Nations; ^4^ ISRIC—International Soil Reference and Information Centre SoilGrids: https://files.isric.org/soilgrids/former/2017-03-10/data/ (accessed on 1 October 2021); ^5^ https://www.resdc.cn/ (accessed on 1 October 2021); ^6^ http://data.cma.cn/ (accessed on 1 October 2021).

**Table 2:** Mean values of slope and terrain relief of different land use regions.

	Cropland	Grassland	Forest	Bare Land
Slope	2.534	5.729	6.672	12.096
Terrain relief	115.965	165.528	203.742	39.893

**Table 3:** Mean values of E_R_K_ of soil types that have a greater impact.

	RFS	RFP	K_cf_	K_f–cs_	St
Leptosols	24.317	25.522	0.0018	0.0039	0.423
Cryosols	23.290	24.311	0.0015	0.0037	0.513
Gypsisols	20.275	19.159	0.0018	0.0027	0.329
Solonchaks	19.942	20.556	0.0019	0.0028	0.347
Gypsisols	18.535	17.356	0.0011	0.0025	0.286
Luvisols	17.017	18.359	0.0011	0.0016	0.307
Calcisols	16.717	17.256	0.0010	0.0016	0.267

**Table 4:** Mean values of E_R_K_ of soil types that have a smaller impact.

Soil Type	RFS	RFP	K_cf_	K_f–cs_	St
Fluvisols	6.667	7.203	0.0006	−0.0002	0.122
Phaeozems	7.800	8.762	0.0006	−0.0001	0.122
Chernozems	11.852	12.883	0.0007	0.0010	0.103
Kastanozems	12.076	14.126	0.0009	0.0003	0.206
Cambisols	12.180	13.039	0.0007	−0.0007	0.125
Acrisols	12.777	13.651	0.0008	−0.0002	0.179
Cambisols	13.274	14.338	0.0009	0.0006	0.234
Alisols	16.949	17.847	0.0010	−0.0002	0.256

## Data Availability

All data, models, and code that support the findings of this study are available from the corresponding author upon reasonable request.
